# User Evaluation of the Effects of a Text Simplification Algorithm Using Term Familiarity on Perception, Understanding, Learning, and Information Retention

**DOI:** 10.2196/jmir.2569

**Published:** 2013-07-31

**Authors:** Gondy Leroy, James E Endicott, David Kauchak, Obay Mouradi, Melissa Just

**Affiliations:** ^1^Information Systems and TechnologyClaremont Graduate UniversityClaremont, CAUnited States; ^2^Eller College of ManagementDepartment of Management Information SystemUniversity of ArizonaTucson, AZUnited States; ^3^Computer Science DepartmentMiddlebury CollegeMiddlebury, VTUnited States; ^4^Rutgers University LibrariesRutgers, The State University of New JerseyNew Brunswick, NJUnited States

**Keywords:** text simplification, health literacy, consumer health information, natural language processing, evaluation study

## Abstract

**Background:**

Adequate health literacy is important for people to maintain good health and manage diseases and injuries. Educational text, either retrieved from the Internet or provided by a doctor’s office, is a popular method to communicate health-related information. Unfortunately, it is difficult to write text that is easy to understand, and existing approaches, mostly the application of readability formulas, have not convincingly been shown to reduce the difficulty of text.

**Objective:**

To develop an evidence-based writer support tool to improve perceived and actual text difficulty. To this end, we are developing and testing algorithms that automatically identify difficult sections in text and provide appropriate, easier alternatives; algorithms that effectively reduce text difficulty will be included in the support tool. This work describes the user evaluation with an independent writer of an automated simplification algorithm using term familiarity.

**Methods:**

Term familiarity indicates how easy words are for readers and is estimated using term frequencies in the Google Web Corpus. Unfamiliar words are algorithmically identified and tagged for potential replacement. Easier alternatives consisting of synonyms, hypernyms, definitions, and semantic types are extracted from WordNet, the Unified Medical Language System (UMLS), and Wiktionary and ranked for a writer to choose from to simplify the text. We conducted a controlled user study with a representative writer who used our simplification algorithm to simplify texts. We tested the impact with representative consumers. The key independent variable of our study is lexical simplification, and we measured its effect on both perceived and actual text difficulty. Participants were recruited from Amazon’s Mechanical Turk website. Perceived difficulty was measured with 1 metric, a 5-point Likert scale. Actual difficulty was measured with 3 metrics: 5 multiple-choice questions alongside each text to measure understanding, 7 multiple-choice questions without the text for learning, and 2 free recall questions for information retention.

**Results:**

Ninety-nine participants completed the study. We found strong beneficial effects on both perceived and actual difficulty. After simplification, the text was perceived as simpler (*P*<.001) with simplified text scoring 2.3 and original text 3.2 on the 5-point Likert scale (score 1: easiest). It also led to better understanding of the text (*P*<.001) with 11% more correct answers with simplified text (63% correct) compared to the original (52% correct). There was more learning with 18% more correct answers after reading simplified text compared to 9% more correct answers after reading the original text (*P*=.003). There was no significant effect on free recall.

**Conclusions:**

Term familiarity is a valuable feature in simplifying text. Although the topic of the text influences the effect size, the results were convincing and consistent.

## Introduction

### Background and Significance

Text is an important source for health-related information. It is easy to create, maintain, and distribute, and medical practitioners often use it to provide instructions and details on treatments. Health-related text is becoming increasingly available with an estimated 80% of online users [1] from a wide array of backgrounds [2] using the Internet to obtain health-related information. The information itself is diverse and includes prevention, treatment, and management of diseases and comes from a variety of sources ranging from professionals to salespeople to patients.

Unfortunately, 90 million Americans have difficulty understanding and acting upon health information [3], and many find the text currently available difficult to read [4]. Some of this difficulty can be attributed to inherent complexity in understanding the diseases, their causes, and the associated treatments, which may require advanced knowledge of biology, chemistry, or physiology to understand in detail. Much of the difficulty, though, can be attributed to a mismatch between the content delivered and the consumers who often have limited health literacy, low general education, or inadequate language skills. Low health literacy reduces health statuses of individuals [3], is considered a “silent killer” [5], and is estimated to cost up to US$238 billion annually [6].

To increase health literacy, the method, medium, and language used play an important role. While one-on-one teaching may be the best solution, medical professionals do not have sufficient time or resources for this. Video and interactive methods can be very educative and are becoming increasingly available. The power of such methods to teach and demonstrate will likely play an important role in consumer health information. However, currently text remains the primary tool used to educate people.

### Factors Influencing Text Difficulty and Its Measurement

**Figure 1:**
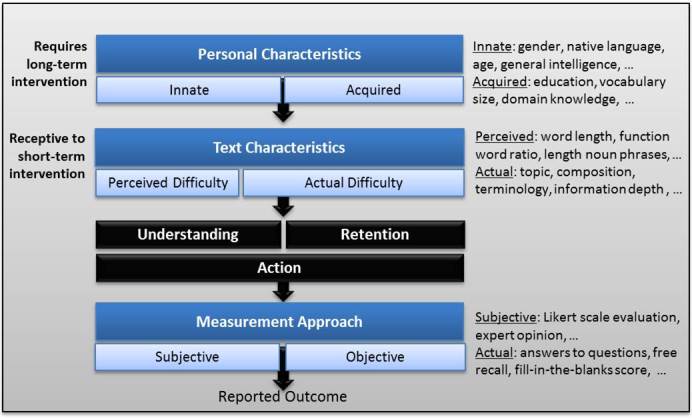
Factors that influence understanding and retention of information.

Figure 1 provides an overview of three key factors representing the authors’ view on influences on understanding and learning from text: personal characteristics, text characteristics, and measurement characteristics. Personal characteristics describe attributes about the reader. Some are innate and cannot be changed, for example, native language and general intelligence. Others are acquired, for example, vocabulary size and domain knowledge. Many of these characteristics have a direct effect on text comprehension and indirectly on learning since comprehension has been shown to affect learning [7]. For example, stress, a personal characteristic, has been shown to affect reading behaviors. People with high stress rely more on visual summaries, even when incomplete, to answer text-based questions [8]. Moreover, increased stress has also been related to lower comprehension of medical terminology [9]. Other personal characteristics, such as the ability to form a good mental model, affects understanding since readers often rely on the mental model instead of the original text base [10]. In addition, past behaviors and acquired skills can have an impact. Exposure to print, for example, has been found to be related to understanding. Landi [11] found a positive relation with results for an author recognition test [12] and question-answering tasks, while in our own work, we found a positive relation between self-reported reading and results for a fill-in-the-blank Cloze test [13].

Text characteristics influence text difficulty and therefore understanding. These characteristics can be adjusted to improve the usefulness of text, but this has been shown to be challenging and very few studies have shown strong improvements in reader understanding. To further clarify the analysis of text characteristics and the text simplification problem in general, we distinguish between the perceived and actual text difficulty of a text. The distinction is based on evidence for the existence of perceived barriers from the Health Belief Model [14] and the importance of perceived difficulty of behavioral control from the Theory of Planned Behavior [15]. While actual difficulty is easily accepted as important, perceived difficulty cannot be ignored. At a minimum, it will impact whether or not a text will be read. However, it may affect health literacy in more ways; for example, Velayo [16] found that a higher perceived difficulty correlated with a decrease in the recall of information.

Text characteristics can include surface features, for example, spacing or font, and range from smaller units such as words, to larger units such as sentences or paragraphs. Using a Likert scale to measure perceived difficulty, it was found that texts with a higher ratio of function words, verbs, verb phrases, or containing more high-frequency words were seen as easier [17-19]. For actual difficulty, simple surface features such as font and line spacing were shown not to affect remembering [20]; however, using a fill-in-the-blanks test additive and causal connectors were shown to be easier than adversative or sequential connectors [21]. In addition to surface features, analysis can include broader features such as coherence, which is defined as good flow combined with a structured, logical argument [22,23]. We found that increasing coherence with proper spacing around subtopics and better logical connectors improved question-answering (actual difficulty) [13]. Not surprisingly, how a topic is presented in a text also influences learning; topics introduced as part of a refutation text, a text where misconceptions are explicitly addressed, led to increased learning and more valid inference but not increased quantity of information being recalled [7].

*Measurement characteristics* also play an important role in readability research, although they are often ignored. Historically, the most popular measurement has been readability formulas, which generate a single number often based only on relative word and sentence length and are used as stand-ins for text complexity [24]. These formulas have become popular even though they ignore current knowledge about the reading process, have a shaky statistical basis, and are unhelpful as writing guidelines [25]. The Flesch-Kincaid Grade Level formula is the most common in health care literature [26]. Even though different tools using the formula sometimes return different levels for the same text [27], it has been used to evaluate patient education materials [28], general websites [29], and information on specific topics such as abdominal aortic aneurysms [30] and back pain [31]. Other readability formulas, such as the Simple Measure of Gobbledygook (SMOG) and Gunning Fog Index, have also been shown to be problematic for evaluating health-related materials for similar reasons [32]. Simplifying text based on these formulas sometimes results in more difficult text, that is, the simplicity paradox [5], because the simplification concentrates on writing style rather than content [2]. As a result, increasingly more concerns are raised about the effectiveness of these formulas for simplifying consumer health texts [33].

Better measures should be developed and used to evaluate text and motivate algorithmic components. These must be evaluated on a representative sample and measure not just the perception of difficulty, but more importantly understanding and retention of information. By using different measures, we can better evaluate the impact of simplification tools. For example, question-answering tasks (eg, multiple-choice, open-ended, or free recall questions), fill-in-the blanks tasks (eg, multiple-choice or open blanks) and teach-back methods (eg, explain a concept or summarize a topic) can be used to measure understanding. Measures that test retention of information can follow the same style of questions, while measures of learning from a text require a comparison between pre- and post-reading scores.

Interactions can also exist between personal, text, and even measurement characteristics. For example, the impact of text coherence on the reader has been found to interact with user characteristics and with the type of measurement. Overall coherence did not affect recall (actual difficulty) but affected remembering and understanding when measured by question-answering (actual difficulty) for readers with high knowledge but low interest or low knowledge but high interest in a topic [10,22]. Personal interest in the topic has also repeatedly been shown to be relevant. A higher interest leads to increased learning [34] and recall [35], however, the coherence of text [34] and prior knowledge [35] influence this relationship.

### Objective

Our objective is twofold. First, we address the need for an evidence-based algorithm that pinpoints difficult text. Second, we focus on providing appropriate, easier alternatives to a writer in an effective and efficient manner. We present here our first fully automated version of the *lexical* simplification algorithm, which identifies difficult terms and generates a list of easier alternatives based on information extracted from dictionaries and other databases. In a pilot study [36], we introduced the text simplification algorithm and presented an initial user study. This work builds upon the lessons learned in the pilot study and differs in a number of key dimensions: (1) the algorithm examined here is fully automated, (2) the simplification of text is done by an independent writer, not the developers, and (3) the evaluation is based on a new study with different participants, new stimuli, and new more comprehensive metrics.

## Methods

### Text Simplification Algorithm and Writing Process

The automated algorithm executes two steps. The first step is *identification of difficult terms.* We conducted corpus analyses and found that the term familiarity differed between easy and difficult texts [17,18]. Motivated by this, our algorithm uses the Google Web Corpus [37], which contains *n*-gram counts from a corpus of 1 trillion words from public webpages to identify difficult terms. Terms with a low frequency in this corpus are assumed to be less familiar and therefore more difficult since a reader would not encounter them often. We used unigrams and the 5000^th^ most frequent word, which has a frequency of 15,377,914, as our threshold for distinguishing less familiar terms. Any term with a lower frequency is considered difficult and is a candidate for replacement.

We used the Google Web Corpus because its terms are representative of everyday readers without special medical knowledge. Other resources may provide additional value but may also introduce inconsistencies. For example, the Google Book Corpus contains many medical books resulting in higher frequencies for medical terms. The Unified Medical Language System (UMLS) contains both medical and general terms. Distinguishing between them algorithmically would be necessary, which is not an easy task, and may not improve upon the frequency-based approach by much.

The second step is the *identification and presentation of easier alternatives for each difficult term*. The list of candidate replacements is generated from synonyms and hypernyms from WordNet 2.0 [38,39]; definitions and semantic types from the UMLS; and definitions from both the English and Simple English Wiktionaries. Only alternatives that possess the same part of speech based on an automatic tagger are presented. In addition, only substitutions with a higher term frequency than the original word are suggested (ie, more familiar). The number of alternatives provided can be adjusted based on user preference or application; currently, we aim to provide a minimum of 7 alternatives. Candidate replacements are sorted both by source (for the convenience of the writer) and by their familiarity in the Google Web Corpus.

In contrast to the previous version of our simplification algorithm [36], which involved one of the authors manually looking up each word to generate the candidate suggestions, the current version is fully automated. To ensure that the algorithm is sufficiently efficient for later inclusion in a comprehensive tool, we tested its efficiency on Wikipedia articles. We selected 100 conditions randomly (see Multimedia Appendix 1) from a list of diseases provided by the Mayo Clinic. For each disease, we retrieved the corresponding Wikipedia article. The articles were on average 2573 words long. On average, 617 words were tagged as difficult per article, for which easier alternatives were produced by the algorithm where available. The average run time was 37 seconds per document.

Given the difficulty of completely automated translation, especially in domains such as health where information may not be omitted, we require a writer to finalize the text. At present, a Microsoft Excel spreadsheet is generated containing each original sentence from a text, the same sentence with blanks for all difficult words, and alternatives for each difficult word. The alternatives are presented in a column and ordered according to source and term familiarity. The writer chooses the best alternative, replacing it in the original text. Ensuring grammatical correctness (eg, consistent pluralization) is currently the responsibility of the writer.

### Original and Simplified Texts (Study Stimuli)

A subject expert (SE), a medical librarian, simplified the texts. To optimize external validity, we worked with one expert to rewrite the text since this is how the final tool will be used. To increase internal validity, we provided the SE with rules to ensure that we measured only the effects resulting from interaction with our algorithm. She was asked to “Try to replace as many words as possible” and when making a replacement “single words can just be replaced but longer fragments should be added before or after the sentence (with some adjustment for flow of text*)*”. The SE served two main roles: (1) to determine if a difficult word flagged by the algorithm needs to be replaced, and (2) for those words requiring replacement, to select an appropriate substitution from the alternatives suggested by the algorithm. If the SE deemed that an appropriate synonym existed for a difficult word in the algorithmically generated options, then the difficult word was simply replaced by the synonym. If the simplification option selected by the SE was not a synonym, it needed to be added to the text so that no original information was deleted from the text. Simplifications containing longer phrases or sentences (eg, from definitions) were added by using parentheses or by adding a separate sentence before or after the target sentence. The text was adjusted by the SE as necessary to create grammatically correct sentences.

In previous work [36], we noticed that lexical simplifications by the authors reduced the flow of the text thereby increasing text difficulty. Therefore, the SE was asked to pay close attention to how alternatives were inserted and to choose the option that resulted in the best flow. If the SE preferred a term other than those suggested by the algorithm, she could add it to the text for familiarity verification. Once the text was rewritten, it was rerun through the simplification algorithm to ensure that newly added text was sufficiently simple. This included the verification of any synonyms by the SE.

To measure *perceived difficulty*, we selected 5 text snippets; these were individual sentences and in one case 2 short sentences combined. Such short snippets do not require much time to read, provide more data points than one long text, and ensure that study participants do not get overwhelmed. The sentences were taken from English Wikipedia articles, and each sentence was simplified by the SE using our algorithm. Our algorithm tagged an average of 11 words per sentence as difficult, of which 5.6 (53%) were replaced.

To measure *actual difficulty,* it was necessary to use longer texts to allow for questions about the content to be posed. We used two different texts so that each participant in the study worked with an original and simplified text for better (statistical) control of interpersonal differences. We chose a text on liver cirrhosis and one on asthma because most people are somewhat familiar with them and both conditions have several commonly accepted myths associated with them. These myths were incorporated into our multiple-choice questions and provided an excellent opportunity to demonstrate learning. Each text was simplified using our approach described above. Texts were obtained from the initial summary paragraphs from their Wikipedia Web pages and were similar in composition. Our algorithm tagged 210 words as difficult in the liver cirrhosis document, of which 66 (31%) were replaced by the writer during simplification. In the asthma document, 122 words were tagged as difficult and 53 (43%) were replaced during simplification.

Tables 1 and 2 show an overview of the text characteristics before and after simplification. We include the Flesch-Kincaid Grade Level for comparison with other work. Below are examples of an original and simplified snippet used as part of the study (perceived difficulty):

- original: “Gout is a disorder of purine metabolism, and occurs when its final metabolite, uric acid, crystallizes in the form of monosodium urate, precipitating in joints, on tendons, and in the surrounding tissues.”
- simplified: “Gout is a disease of the processing of the chemical substance called purine, and occurs when its last chemical product (uric acid) makes crystals (monosodium urate), which collect in joints, on tendons, and in the surrounding tissues.”

The texts, both original and simplified versions, are provided in Multimedia Appendix 2.

**Table 1:** Text snippet characteristics.

	Lexical simplification	
Sentences (N=5)	Original	Simplified
Word count (avg)	28.4	37.6
Flesch-Kincaid grade level (avg)	18.6	17.3

**Table 2:** Document characteristics.

	Lexical simplification					
Documents	Original			Simplified		
Topic	Asthma	Liver cirrhosis	Average	Asthma	Liver cirrhosis	Average
Word count	623	481	552	779	696	737.5
Sentence count	31	25	28	33	27	30
Flesch-Kincaid grade level	13.9	14.5	14.2	13.7	14.3	14.0

### Metrics

To measure *perceived difficulty*, participants judged a sentence using a 5-point Likert scale with the following labels: Very Easy, Easy, Neither, Hard, Very Hard. Perceived difficulty is the score on this scale with 1 representing Very Easy and 5 Very Hard.

To measure *actual difficulty*, we used metrics covering understanding, learning, and retention of information. For understanding of the text, we used 5 multiple-choice questions posed alongside the text. The questions targeted different sections of the text. Understanding was measured as the percentage of questions answered correctly.

To measure learning, we compared scores on 7 multiple-choice questions shown both before and after reading the text. The text itself was not visible when the questions were presented. By asking the same questions before and after, we were able to use participants as their own controls. For each text, we created the multiple-choice questions based on commonly accepted myths. The myths were gathered by searching the Internet for “common myths about…”. Learning was measured as the increase in the percentage of questions answered correctly after versus before reading the text.

To measure retention, we asked participants after all sections have been completed to list all facts (one per line) that they remembered from the texts. Retention can be simply measured as the number of facts listed, however, since these facts may contain errors, they were also graded by the authors. Two authors per topic independently graded all facts. Even though participants were asked to list 1 fact per line, many lines included multiple facts per line. Each fact was considered and awarded points separately: +1 for a correct fact and -1 for an incorrect. To grade the answers in an objective manner, the order of answers was randomized per grader and the experimental condition unknown. In cases with a large disparity between grades (scores diverged by more than 100%), a third grader (the SE) judged the results and provided the final score (similar to original manual GRE scoring [40]). Retention was then measured with 2 metrics: the number of listed facts and the sum of the grades assigned to those facts.

In addition to study questions, we also included qualifying questions. These were simple questions for which the answer was obvious. They helped filter results of participants who were not serious about the study. We included a qualifying question with each set of multiple-choice questions and filtered any participant who did not get all qualifying questions right.

### Participants

Participants were recruited using Amazon’s Mechanical Turk. MTurk is an online crowdsourcing service that allows for small tasks to be accomplished by human workers. Currently, Amazon has over 300,000 requested tasks and over half a million workers. Workers are paid a small sum for each task accomplished. MTurk has been used in a wide range of settings ranging from user studies to data annotation to subjective rating generation [41]. The workers are a diverse group from all over the world with varied demographic characteristics [42,43]. When precautions are taken to filter out ineffective workers, the quality of the data obtained has been shown to be at least as good as data obtained from more traditional approaches [43,44].

### Procedure

Participants were directed to our study website from MTurk, and the sections were presented in the following order:

- The *first page* contained the welcome note and instructions to complete the study sections in order and without use of external sources. From this point, the browser back button was disabled.
- The *first study section* showed the myth-based questions for a topic. Then, the text was shown together with new questions, followed by a repetition of the myth-based questions without the text. For each participant, the order of the questions and answers for each question were randomized. The topic was either liver cirrhosis or asthma, and the version was either original or simplified.
- The *second study section* was identical to the first, but with a different text in a different version. Each participant received one original and one simplified version. The order and topics were balanced over the study so that all combinations of topic and difficulty level were presented.
- The *third study section* contained the individual sentences that participants judged for perceived difficulty. The original and simplified version of a sentence were paired because showing all sentences in one list made it very difficult for participants to notice differences and provide a rational judgment. The order within each pair and the order of the 5 pairs were randomized per participant.
- The *fourth study section* contained demographic questions.
- The *fifth and sixth study sections* contained the PSS-10 [45], a standardized stress survey, and the STOFHLA [46,47], a standardized health literacy measure.
- The *seventh and eighth sections* contained the request for free recall of information for the first and second text.
- The *final page* showed a Thank You note and the code to be submitted for payment at MTurk.

## Results

### Participant Characteristics

We invited MTurk workers located in the United States with a 95% approval rate on tasks previously performed for other requesters. They were paid US$1.50 for completing the survey. Upon start, 134 participants signed up and 105 completed the study. Of those who completed, 6 did not pass our filtering criteria resulting in a total of 99 valid participants. Completing the survey took on average 33 minutes. The shortest time spent was 13 minutes and the longest was 45 minutes.

Table 3 provides the demographic information. Most participants (80%) were between 21 and 50 years old, with only a small group younger than 20 (3%) or older than 60 years (4%). The majority were female (63%), white (89%), and not Hispanic or Latino (93%). Most had moderate education: 48% had a high school diploma, 16% an associate’s degree, and 25% a bachelor’s degree. The majority (89%) spoke exclusively English at home.

### Perceived Difficulty

**Figure 2:**
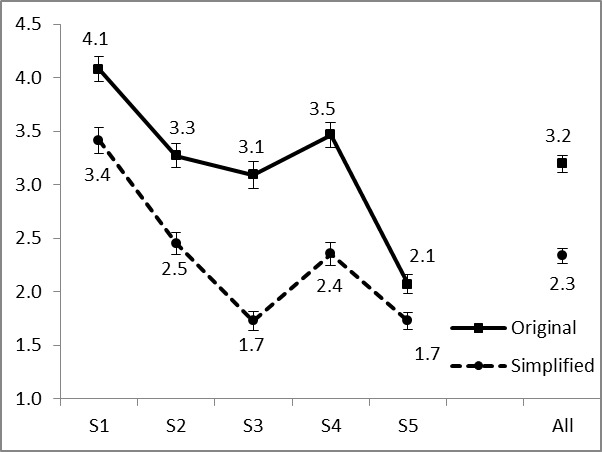
Average perceived difficulty scores (lower score = perceived simpler).

We found a significant beneficial effect of simplification on perceived difficulty with simplified sentences being judged as simpler. Figure 2 shows an overview of the average score and standard error bars for each sentence and for all sentences combined. A paired-samples *t*-test showed the difference to be significant for all pairs (*P*<.001) and for all pairs combined (*P*<.001).

**Table 3:** Participant demographic information (n=99).

Characteristics		n
**Age**		
	20 or younger	3
	21-30	35
	31-40	24
	41-50	21
	51-60	12
	61-70	4
	71 or older	-
**Gender**		
	Female	62
	Male	37
**Race (multiple choices allowed)**		
	American Indian / Native Alaskan	2
	Asian	7
	Black or African American	5
	Native Hawaiian or Other Pacific Islander	-
	White	88
**Ethnicity**		
	Hispanic or Latino	7
	Not Hispanic or Latino	92
**Education (highest completed)**		
	Less than High School	1
	High School Diploma	48
	Associate’s Degree	16
	Bachelor’s Degree	25
	Master’s Degree	6
	Doctorate	3
**Language skills (frequency of speaking English at home)**		
	Never English	-
	Rarely English	1
	Half English	3
	Mostly English	6
	Only English	89

### Actual Difficulty: Understanding, Learning, and Retention

**Figure 3:**
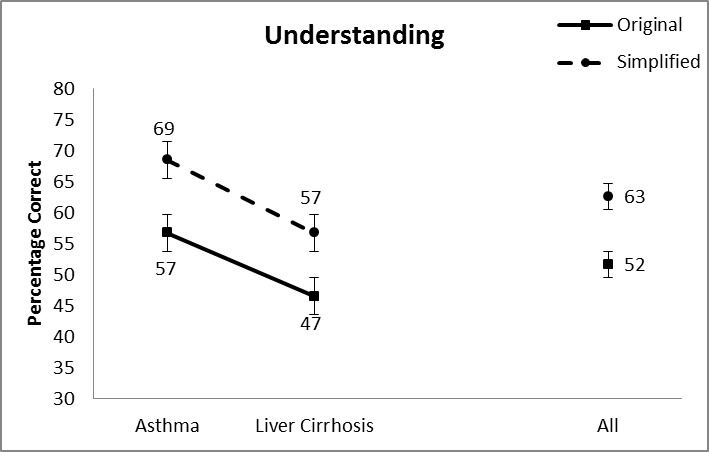
Average understanding scores.

Figure 3 shows the mean scores and standard error bars for understanding. We conducted a two-way analysis of variance (ANOVA) with simplification and topic as independent variables. Topic was included to provide a more nuanced view. For understanding, we found two main effects. The first is for simplification with higher scores for simplified text. There were on average 52% correct answers with an original document and 63% with a simplified document, (*F*_1,198_=13.869, *P*<.001). There was also a main effect for topic (*F*_1,198_=13.869, *P*<.001) with higher scores achieved for the asthma document. Since the increases in understanding after simplification were comparable for both topics, the interaction effect was not significant.

**Figure 4:**
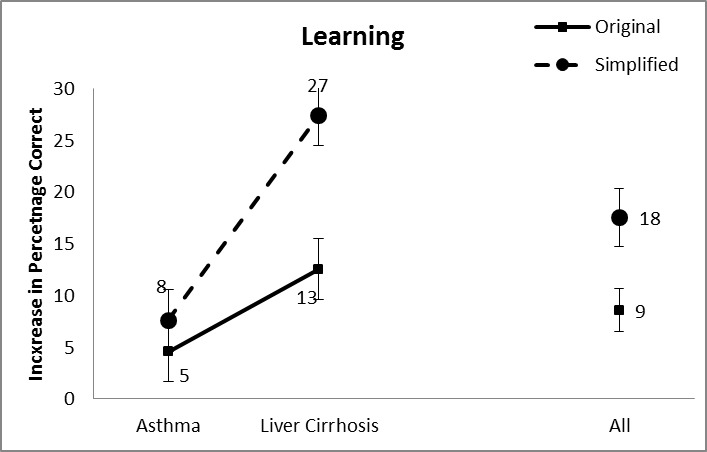
Average learning scores.

Figure 4 shows the mean scores and standard error bars for the learning of information. We conducted a comparable two-way ANOVA with the simplification and topic as independent variables. We found a significant main effect of simplification of text with more learning from simplified documents (18%) than from the original documents (9%) (*F*_1,198_=9.238, *P*=.003). A second main effect was found for topic (*F*_1,198_=22.301, *P*<.001) with more learning with the liver cirrhosis document (20%) than with the asthma document (6%). The interaction between both independent variables was also significant (*F*_1,198_=4.071, *P*=.045) with the learning being more pronounced with the liver cirrhosis than with the asthma document.

Table 4 provides an overview of the retention of information using both raw and graded scores. With simplified documents, slightly more facts were listed (5.04) than with original documents (4.66). There were also slightly more words (43.60) and unique words (32.36) used after reading simplified documents compared to original documents (40.07 words and 30.79 unique words). These differences were not statistically significant. The graded scores show similar small differences. There were slightly more correct facts after reading simplified documents (5.04 facts) than after reading the original documents (4.70 facts). However, the difference is not statistically significant.

**Table 4:** Retention of information: mean and standard deviation.

	Original text			Simplified text		
Average counts	Asthma	Liver cirrhosis	Average	Asthma	Liver cirrhosis	Average
Facts	4.76	4.55	4.66	4.96	5.12	5.04
Words	40.50	39.63	40.07	42.90	44.24	43.60
Unique words	31.38	30.20	30.79	32.39	32.34	32.36
Average score (graded facts)	4.50	4.91	4.70	4.73	5.35	5.04

### Relationships With Participant Characteristics

To complete our analysis, we conducted a correlation analysis using a 2-tailed Pearson product-moment correlation coefficient (*r*). We evaluated the personal characteristics and the scores for perceived and actual difficulty over experimental conditions. We assigned a code to the education level and language skills with a higher score indicating a higher level or skill. We also included the PSS scores and STOFHLA scores.

Overall, there were few significant correlations. There were no significant correlations between the perceived difficulty of sentences and the personal characteristics. For actual difficulty, only education mattered. There was a positive correlation between education and understanding (*r*=.244, *P*=.015), facts listed (*r*=.296, *P*=.003), graded facts (*r*=.411, *P≤*.001), and both the word count (*r=*.316, *P*=.001) and unique word count (*r*=.329, *P*=.001). Among the personal characteristics themselves, two correlations were significant. There was a negative correlation between language skills and stress levels, indicating higher stress related to lower language skills (*r*=-.210, *P*=.037) and also a negative correlation between language skills and education level (*r*=-.260, *P*=.009). Upon closer inspection, this last negative correlation was due to a few individuals with higher degrees who speak a different language at home, that is, Chinese, Tamil, or Farsi.

## Discussion

### Principal Findings

This work reported on a lexical simplification algorithm that automatically detects difficult terms and suggests easier alternatives. The writing process is semiautomated since the final replacements are made by the writer. A controlled user study showed how simplifying text in this manner led to significant improvements in both perceived and actual difficulty of text.

The results on perceived difficulty corroborate earlier work on manual lexical simplification. In general, changing the text to improve perceived difficulty is more straightforward. Consistent and strong effects are found even when using short text snippets or small sample sizes. Even so, this effect is important and shows that *lexical simplification has a beneficial impact on perceived difficulty*. Future studies will look more closely at how perceived difficulty affects motivation to read and ability to complete reading, among other factors.

The results on actual difficulty are strong and very encouraging. They also show the importance of using different metrics. We found a strong effect on understanding with simplified text being better understood. However, this effect also depended on the topic being studied. Learning showed a similar strong effect: there was more learning with simplified documents. These effects lead to our conclusion that *lexical simplification is beneficial and has an immediate impact on understanding and learning*. However, we did not find an effect of simplification on retention of information. This may be due to a lack of sustained learning or it may be due to the study design. In previous work on search engines [48], we found that many study participants stop finding information at some given point, regardless of how easy or difficult a task is. We may be witnessing a similar effect with participants submitting “enough” facts regardless of how many they remember. In future work, we aim to provide better incentives to encourage participants to submit more facts.

### Limitations

There are several limitations we would like to point out. First, we evaluated our approach with short texts taken from Wikipedia. Different effects may be found for longer or more difficult texts. However, working with short texts allows for a controlled experiment, thereby avoiding potentially confounding variables. Future work will look for repeat effects in longer documents. Second, we worked with general topics. Automatically recognizing which different texts, either distinguished by difficulty level or other factors, would benefit from simplification would be an important addition to our work. In addition, working with personally relevant topics may increase effects, since motivation has been shown to be important to the reading process. Third, we worked with only one subject expert who rewrote text. Comparing different writers may show further strengths and weaknesses of our approach. Working with a team of writers may provide a more balanced gold standard; however, this approach has also been shown to introduce noise when experts disagree [49]. Further research is needed to understand the impact of each of these limitations.

### Conclusions

In addition to these study limitations, there is also much room for future development of our algorithm. We aim to more precisely target difficult words so that fewer words are tagged for replacement while still impacting the overall difficulty of text. We aim to provide a shorter and more precise list of potential replacements by working with resources such as the Consumer Health Vocabulary [50-52]. This will make the process more efficient for the writer while requiring less time to generate alternatives. For example, we plan to test phrases in addition to individual words to estimate difficulty and work with different thresholds. We also are working toward combining lexical simplification with other forms of simplification of relevant text features.

## Multimedia Appendix 1

List of conditions.

## Multimedia Appendix 2

Stimuli: Original and simplified texts.

## Abbreviations

ANOVA: analysis of variance
GRE: Graduate Record Examinations
SE: subject expert
UMLS: Unified Medical Language System

## References

[1] Fox S (2011). Health Topics.

[2] Wang Y (2006). Automatic Recognition of Text Difficulty from Consumers Health Information. Proceedings of the 19th IEEE International Symposium on Computer-Based Medical Systems.

[3] Nielsen-Bohlman L, Institute of Medicine /Committee on Health (2004). ‘Health literacy: a prescription to end confusion’.

[4] Yan X, Song D, Li X (2006). Concept-based Document Readability in Domain Specific Information Retrieval. Proceedings of the 15th ACM International Conference on Information and Knowledge Management.

[5] Zarcadoolas C (2011). The simplicity complex: exploring simplified health messages in a complex world. Health Promot Int.

[6] Low health literacy report.

[7] Diakidoy I-A, Mouskounti T, Ioannides C (2011). Comprehension and Learning from Refutation and Expository Texts. Reading Research Quarterly.

[8] Leroy G, Miller T (2010). Perils of providing visual health information overviews for consumers with low health literacy or high stress. J Am Med Inform Assoc.

[9] Van Servellen G, Brown JS, Lombardi E, Herrera G (2003). Health literacy in low-income Latino men and women receiving antiretroviral therapy in community-based treatment centers. AIDS Patient Care and STDs.

[10] Kintsch W (1986). Learning from Text. Cognition and Instruction.

[11] Landi N (2010). An examination of the relationship between reading comprehension, higher-level and lower-level reading sub-skills in adults. Read Writ.

[12] Stanovich KE, West RF (1989). Exposure to Print and Orthographic Processing. Reading Research Quarterly.

[13] Leroy G, Lauchak D, Mouradi O (2013). A User-study Measuring the Effects of Lexical Simplification and Coherence Enhancement on Perceived and Actual Text Difficulty. Int J Med Inform.

[14] Janz NK, Becker MH (1984). The Health Belief Model: a decade later. Health Educ Q.

[15] Trafimow D, Sheeran P, Conner M, Finlay KA (2002). Evidence that perceived behavioural control is a multidimensional construct: perceived control and perceived difficulty. Br J Soc Psychol.

[16] Velayo RS (1993). Retention of Content as a Function of Presentation Mode and Perceived Difficulty. Reading Improvement.

[17] Leroy G, Endicott J (2011). Term Familiarity to Indicate Perceived and Actual Difficulty of Text in Medical Digital Libraries. Proceedings of the International Conference on Asia-Pacific Digital Libraries (ICADL ) - Digital Libraries -- for Culture Heritage, Knowledge Dissemination, and Future Creation.

[18] Leroy G, Endicott J (2012). Combining NLP with Evidence-based Methods to Find Text Metrics related to Perceived and Actual Text Difficulty. Proceedings of the 2nd ACM SIGHIT International Health Informatics Symposium (ACM IHI).

[19] Pitler E, Nenkova A (2008). Revisiting Readability: A Unified Framework for Predicting Text Quality. Proceedings of the Empirical Methods in Natural Language Processing.

[20] Soleimani H, Mohammadi E (2012). The Effect of Text Typographical Features on Legibility,Comprehension, and Retrieval of EFL Learners. English Language Teaching.

[21] Goldman SR, Murray JD (1992). Knowledge of Connectors as Cohesion Devices in Text: A Comparative Study of Native-English and English-as-a-Second-Language Speakers. Journal of Educational Psychology.

[22] Boscolo P, Mason L (2003). Topic Knowledge, Text Coherence, and Interest: How they Interact in Learning from Instructional Texts. The Journal of Experimental Education.

[23] McNamara DS, Kintsch E, Songer NB, Kintsch W (1996). Are Good Texts Always Better? Interactions of Text Coherence, Background Knowledge, and Levels of Understanding in Learning from Text. Cognition and Instruction.

[24] DuBay WH (2004). The Principles of Readability. Impact Information.

[25] Bruce B, Rubin A, Starr K (1981). Why Readability Formulas Fail. IEEE Transactions on Professional Communication.

[26] Wang L-W, Miller MJ, Schmitt MR, Wen FK (2012). Assessing Readability Formula Differences with Written Health Information Materials: Application, Results, and Recommendations. Research in Social & Administrative Pharmacy.

[27] Sirico LJ (2008). Readability Studies: How Technocentrism Can Compromise Research and Legal Determinations. Villanova University Legal Working Paper Series.

[28] Polishchuk DL, Hashem J, Sabharwal S (2012). Readability of online patient education materials on adult reconstruction Web sites. J Arthroplasty.

[29] Ahmed OH, Sullivan SJ, Schneiders AG, McCrory PR (2012). Concussion information online: evaluation of information quality, content and readability of concussion-related websites. Br J Sports Med.

[30] Bailey M, Coughlin PA, Sohrabi S, Griffin KJ, Rashid ST, Troxler MA, Scott DJ (2012). Quality and readability of online patient information for abdominal aortic aneurysms. J Vasc Surg.

[31] Hendrick PA, Ahmed OH, Bankier SS, Chan TJ, Crawford SA, Ryder CR, Welsh LJ, Schneiders AG (2012). Acute low back pain information online: an evaluation of quality, content accuracy and readability of related websites. Man Ther.

[32] Kim H, Goryachev S, Rosemblat G, Browne A, Keselman A, Zeng-Treitler Q (2007). Beyond Surface Characteristics: A New Health Text-Specific Readability Measurement. AMIA Annu Symp Proc.

[33] Gemoets D, Rosemblat G, Tse T, Logan R (2004). Assessing readability of consumer health information: an exploratory study. Stud Health Technol Inform.

[34] Clinton V, Broek P (2012). Interest, inferences, and learning from texts. Learning and Individual Differences.

[35] Erçetin G (2010). Effects of Topic Interest and Prior Knowledge on Text Recall and Annotation use in Reading a Hypermedia Text in the L2. ReCALL.

[36] Leroy G, Endicott J, Mouradi O, Kauchak D, Just M (2012). Improving Perceived and Actual Text Difficulty for Health Information Consumers using Semi-Automated Methods. Proceedings of the American Medical Informatics Association (AMIA) Fall Symposium.

[37] Web 1T 5-gram Corpus Version 1.1.

[38] Fellbaum C (1998). WordNet: An Electronic Lexical Database.

[39] Miller GA (1995). WordNet: a lexical database for English. Commun. ACM.

[40] How the GRE tests are scored.

[41] Kittur A, Chi E, Suh B (2008). Crowdsourcing User Studies with Mechanical Turk. Proceedings of the SIGCHI Conference on Human Factors in System Computing.

[42] Ross J, Irani L, Silberman MS, Zaldivar A, Tomlinson B (2010). Who are the Crowdworkers? Shifting Demographics in Mechanical Turk. Proceedings of the CHI '10 Extended Abstracts on Human Factors in Computing Systems.

[43] Paolacci G, Changler J, Ipeirotis PG (2010). Running Experiments on Amazon Mechanical Turk. Judgment and Decision-making.

[44] Buhrmester M, Kwang T, Gosling SD (2011). Amazon's Mechanical Turk A New Source of Inexpensive, Yet High-Quality, Data?. Perspectives on Psychological Science.

[45] Cohen S, Kamarck T, Mermelstein R (1983). A Global Measure of Perceived Stress. Journal of Health and Social Behavior.

[46] Nurss JR, Parker RM, Williams MV, Baker DW (1995). Test of Functional Health Literacy in Adults.

[47] Parker RM, Baker DW, Williams MV, Nurss JR (1995). The Test of Functional Health Literacy in Adults: A New Instrument for Measuring Patients’ Literacy Skills. Journal of General Internal Medicine.

[48] Leroy G, Lally A, Chen H (2003). The Use of Dynamic Contexts to Improve Casual Internet Searching. ACM Transactions on Information Systems.

[49] Leroy G, Rindflesch TC (2005). Effects of information and machine learning algorithms on word sense disambiguation with small datasets. Int J Med Inform.

[50] Zeng-Treitler Q, Goryachev S, Tse T, Keselman A, Boxwala A (2008). Estimating consumer familiarity with health terminology: a context-based approach. J Am Med Inform Assoc.

[51] Keselman A, Smith CA, Divita G, Kim H, Browne A, Leroy G, Zeng-Treitler Q (2008). Consumer Health Concepts that do not Map to the UMLS: Where Do They Fit?. Journal of the American Medical Informatics Association.

[52] Keselman A, Logan R, Smith CA, Leroy G, Zeng-Treitler Q (2008). Developing informatics tools and strategies for consumer-centered health communication. J Am Med Inform Assoc.

