# Supplementary material for: User Evaluation of the Effects of a Text Simplification Algorithm Using Term Familiarity on Perception, Understanding, Learning, and Information Retention
Source: J Med Internet Res. 2013 Jul 31;15(7):e144. doi: 10.2196/jmir.2569 (PMC3742397; doi:10.2196/jmir.2569)
Supplement: Supplementary file 1 [file jmir_v15i7e144_app1.pdf]

Acne vulgaris

Wilson's disease

Wolff–Parkinson–White syndrome

A wrinkle

Carpal tunnel syndrome (CTS)

Back pain

Rabies

Acute radiation syndrome (ARS)

Ramsay Hunt syndrome (RHS)

Gastric dumping syndrome

Tachycardia

Raynaud's phenomenon

Reactive airway disease

Reactive arthritis

Reactive attachment disorder (RAD)

Medication overuse headaches (MOH)

Medication overuse headaches (MOH)

red eye

Renal failure

Restless legs syndrome (RLS)

Retinoblastoma (Rb)

retrograde ejaculation

Rett syndrome

Reye's syndrome

Abdominal aortic aneurysm

Rheumatic fever

Rheumatoid arthritis (RA)

Allergic rhinitis

Rickets

Dermatophytosis or ringworm

Road rage

Rosacea

Exanthema subitum

Rotavirus

Rubella

Absence seizures

Ichthyosis vulgaris

Myelofibrosis

Impetigo

Erectile dysfunction (ED)

Cervical incompetence

A stress fracture

Dyspepsia

Infertility

inflammatory bowel disease (

Influenza

Acanthosis nigricans

Inflammatory breast cancer

Ingrown hair

An inguinal hernia

Insomnia

Metabolic syndrome

Intermittent explosive disorder (IED)

Interstitial cystitis

Interstitial lung disease (ILD)

Mesenteric ischemia

Bowel obstruction (or intestinal obstruction)

Achilles tendonitis

An intracranial hemorrhage (ICH)

Intraductal carcinoma

Intrahepatic cholestasis of pregnancy

An intussusception

Invasive lobular carcinoma

Uveitis

Iron-deficiency anemia

iron overload

Overactive bladder (OAB)

Irritable bowel syndrome

The Achilles tendon

Ischemic colitis

Tachycardia

Takayasu's

Takotsubo cardiomyopathy

Tapeworm Infestation

Tuberculosis

Bruxism

Giant-cell arteritis

An epileptic seizure

Lateral epicondylitis or lateral epicondylalgia

Gastroesophageal reflux disease (GERD)

A tension headache

Testicular cancer

Testicular torsion

Tetanus

Tetralogy of Fallot (TOF)

Thalassemia

thoracic aortic aneurysm

Thoracic outlet syndrome (TOS)

Rubella

anterior cruciate ligament (ACL)

Tinea cruris

wart

Obesity

West Nile virus (WNV)

Wheat allergy

Whiplash

Whipple's disease

White coat hypertension

Pertussis

Wilms' tumor or nephroblastoma
