# Supplementary material for: User Evaluation of the Effects of a Text Simplification Algorithm Using Term Familiarity on Perception, Understanding, Learning, and Information Retention
Source: J Med Internet Res. 2013 Jul 31;15(7):e144. doi: 10.2196/jmir.2569 (PMC3742397; doi:10.2196/jmir.2569)
Supplement: Supplementary file 2 [file jmir_v15i7e144_app2.pdf]

Original Text Snippets

1. Some other noted consequences of repeated seizures are neuronal loss, gliosis, parenchymal microhemorrhages, excess of starch bodies, leptomeningeal thickening, subpial gliosis, perivascular gliosis and periavascular atrophy.

2. If mild persistent disease is present (more than two attacks a week), low-dose inhaled glucocorticoids or alternatively, an oral leukotriene antagonist or a mast cell stabilizer is recommended.

3. For those who suffer daily attacks, a higher dose of inhaled glucocorticoid is used. In a severe asthma exacerbation, oral glucocorticoids are added to these treatments.

4. Gout is a disorder of purine metabolism, and occurs when its final metabolite, uric acid, crystallizes in the form of monosodium urate, precipitating in joints, on tendons, and in the surrounding tissues.

5. Gout (also known as podagra when it involves the big toe) is a medical condition usually characterized by recurrent attacks of acute inflammatory arthritis-a red, tender, hot, swollen joint.

Simplified Text Snippets

1. Some other important effects of repeated seizures are loss of neurons, gliosis (or too many non-neuronal cells in damaged areas of the central nervous system), parenchymal micro bleedings, too many starch bodies, thickening of the thin layers that cover and protect the brain and spinal cord, subpial gliosis, gliosis and weakening around the veins .

2. If mild repetitive disease is present (more than two attacks a week), low-dose steroids inhaled into the lungs or else an oral (by mouth) medicine called a leukotriene antagonist or a mast cell stabilizer is recommended.

3. For those who have daily attacks, a higher dose of steroids inhaled into the lungs is used. If the severity of the asthma increases, oral (by mouth) steroids are added to these treatments.

4. Gout is a disease of the processing of the chemical substance called purine, and occurs when its last chemical product (uric acid) makes crystals (monosodium urate), which collect in joints, on tendons, and in the surrounding tissues.

5. Gout (also known as podagra when it involves the big toe) is a disease characterized by repeated attacks of sudden inflammatory arthritis - a red, tender, hot, swollen joint.

Original Document: ASTHMA

Asthma (from the Greek άσθμα, ásthma, "panting") is the common chronic inflammatory disease of the airways characterized by variable and recurring symptoms, reversible airflow obstruction, and bronchospasm. Symptoms include wheezing, coughing, chest tightness, and shortness of breath. Asthma is clinically classified according to the frequency of symptoms, forced expiratory volume in 1 second (FEV1), and peak expiratory flow rate. Asthma may also be classified as atopic (extrinsic) or non-atopic (intrinsic).

It is thought to be caused by a combination of genetic and environmental factors. Treatment of acute symptoms is usually with an inhaled short-acting beta-2 agonist (such as salbutamol). Symptoms can be prevented by avoiding triggers, such as allergens and irritants, and by inhaling corticosteroids. Leukotriene antagonists are less effective than corticosteroids and thus less preferred.

Its diagnosis is usually made based on the pattern of symptoms and/or response to therapy over time. The prevalence of asthma has increased significantly since the 1970s. As of 2010, 300 million people were affected worldwide. In 2009 asthma caused 250,000 deaths globally. Despite this, with proper control of asthma with step down therapy, prognosis is generally good.

Causes

Asthma is caused by environmental and genetic factors. These factors influence how severe asthma is and how well it responds to medication. The interaction is complex and not fully understood.

Studying the prevalence of asthma and related diseases such as eczema and hay fever have yielded important clues about some key risk factors. The strongest risk factor for developing asthma is a history of atopic disease; this increases one's risk of hay fever by up to 5× and the risk of asthma by 3–4×. In children between the ages of 3–14, a positive skin test for allergies and an increase in immunoglobulin E increases the chance of having asthma. In adults, the more allergens one reacts positively to in a skin test, the higher the odds of having asthma.

Because much allergic asthma is associated with sensitivity to indoor allergens and because Western styles of housing favor greater exposure to indoor allergens, much attention has focused on increased exposure to these allergens in infancy and early childhood as a primary cause of the rise in asthma. Primary prevention studies aimed at the aggressive reduction of airborne allergens in a home with infants have shown mixed findings. Strict reduction of dust mite allergens, for example, reduces the risk of allergic sensitization to dust mites, and modestly reduces the risk of developing asthma up until the age of 8 years old. However, studies also showed that the effects of exposure to cat and dog allergens worked in the converse fashion; exposure during the first year of life was found to reduce the risk of allergic sensitization and of developing asthma later in life.

The inconsistency of this data has inspired research into other facets of Western society and their impact upon the prevalence of asthma. One subject that appears to show a strong correlation is the development of asthma and obesity. In the United Kingdom and United States, the rise in asthma prevalence has echoed an almost epidemic rise in the prevalence of obesity. In Taiwan, symptoms of allergies and airway hyper-reactivity increased in correlation with each 20% increase in body-mass index. Several factors associated with obesity may play a role in the pathogenesis of asthma, including decreased respiratory

function due to a buildup of adipose tissue (fat) and the fact that adipose tissue leads to a pro-inflammatory state, which has been associated with non-eosinophilic asthma.

Asthma has been associated with Churg–Strauss syndrome, and individuals with immunologically mediated urticaria may also experience systemic symptoms with generalized urticaria, rhino-conjunctivitis, orolaryngeal and gastrointestinal symptoms, asthma, and, at worst, anaphylaxis. Additionally, adult-onset asthma has been associated with periocular xanthogranulomas.

Simplified Document: ASTHMA

Asthma (from the Greek word for "panting") is the common chronic inflammatory disease of the respiratory tract (the passages through which air enters and leaves the body) characterized by variable and repeating symptoms, air flow that is blocked but can be reversed, and bronchospasm. Bronchospasms are muscle cramps of the large air passages between the trachea and the lungs that make breathing out difficult and noisy. Symptoms include wheezing (breathing with a whistling sound), coughing, chest tightness, and being short of breath. Asthma is medically classified according to the frequency of symptoms, the amount of air you can force while breathing out in 1 second (called FEV1), and the rate at which you breathe out (called peak expiratory flow rate). Asthma may also be classified as atopic (caused by an allergic reaction) or non-atopic (when the cause is not known).

It is thought to be caused by a combination of genetic and environmental factors. Treatment of acute symptoms, those that begin and get worse quickly, is usually with an inhaled medicine that is breathed into the lungs. One inhaled medication is called salbutamol and it works as a short-acting beta-2 agonist. Symptoms can be kept from happening by staying away from triggers, such as things you are allergic to and irritants, and by inhaling medicines called corticosteroids. Another kind of medicine called leukotriene antagonists is not as effective as corticosteroids so it is less preferred.

Its diagnosis is usually made based on the pattern of symptoms and/or response to therapy over time. The number of asthma cases has increased significantly since the 1970s. As of 2010, 300 million people were affected worldwide. In 2009 asthma caused 250,000 deaths around the world. Despite this, with the proper control of asthma with step down therapy, the medical outcome is generally good.

Causes

Asthma is caused by environmental and genetic factors. These factors influence how severe asthma is and how well it reacts to medication. The interaction is complex and not fully understood.

Studying the number of asthma cases and related diseases such as eczema (inflammation of the skin) and hay fever (allergies to trees, weeds, and grasses) have given important clues about some key risk factors. The biggest risk factor for developing asthma is a history of atopic (or allergic) disease; this increases one's risk of hay fever by up to 5 times and the risk of asthma by 3–4 times. In children between the ages of 3–14, a positive skin test for allergies and an increase in immunoglobulin E (which has been associated with allergic sensitivity) increases the chance of having asthma. In adults, the more allergens one reacts positively to in a skin test, the higher the odds of having asthma.

Because much allergic asthma is associated with sensitivity to indoor allergens and because Western styles of housing favor greater exposure to indoor allergens, much attention has focused on increased

exposure to these allergens in infancy and early childhood as a primary cause of the rise in asthma. Primary prevention studies aimed at the aggressive reduction of airborne allergens in a home with babies have shown mixed results. Strict reduction of dust mite allergens, for example, cuts the risk of allergic sensitization to dust mites, and modestly reduces the risk of developing asthma up until the age of 8 years old. However, studies also showed that the effects of exposure to cat and dog allergens worked in the opposite fashion; exposure during the first year of life was found to reduce the risk of allergic sensitization and of developing asthma later in life.

The inconsistency of this data has inspired research into other aspects of Western society and their impact upon the number of asthma cases. There seems to be a relationship between obesity and the chance of developing asthma. In the United Kingdom and United States, the rise in the number of asthma cases has matched a widespread rise in the number of obese people. In Taiwan, symptoms of allergies and airway hyper-reactivity increased in parallel with each 20% increase in body-mass index (a measure of obesity). Several factors linked to obesity may play a role in the development of asthma, including decreased respiratory function due to a buildup of adipose tissue (fat) and the fact that fatty tissue leads to a pro-inflammatory state, which has been associated with non-eosinophilic asthma.

Asthma has been associated with Churg–Strauss syndrome, and individuals with immunologically mediated urticaria (skin rashes) may also experience whole body symptoms with widespread skin rashes, rhino-conjunctivitis, orolaryngeal and gastrointestinal symptoms, asthma, and, at worst, anaphylaxis (a sever and sometimes life-threatening allergic reaction). Additionally, asthma developed in adulthood has been associated with periocular xanthogranulomas.

Original Document: CIRRHOSIS

Cirrhosis is a consequence of chronic liver disease characterized by replacement of liver tissue by fibrosis, scar tissue and regenerative nodules (lumps that occur as a result of a process in which damaged tissue is regenerated), leading to loss of liver function. Cirrhosis is most commonly caused by alcoholism, hepatitis B and C, and fatty liver disease, but has many other possible causes. Some cases are idiopathic (i.e., of unknown cause).

Ascites (fluid retention in the abdominal cavity) is the most common complication of cirrhosis, and is associated with a poor quality of life, increased risk of infection, and a poor long-term outcome. Other potentially life-threatening complications are hepatic encephalopathy (confusion and coma) and bleeding from esophageal varices. Cirrhosis is generally irreversible, and treatment usually focuses on preventing progression and complications. In advanced stages of cirrhosis the only option is a liver transplant.

The word "cirrhosis" derives from Greek κιρρός [kirrhós] meaning yellowish, tawny (the orange-yellow colour of the diseased liver) + Eng. med. suff. -osis. While the clinical entity was known before, it was René Laennec who gave it the name "cirrhosis" in his 1819 work in which he also describes the stethoscope.

Pathophysiology

The liver plays a vital role in synthesis of proteins (e.g., albumin, clotting factors and complement), detoxification and storage (e.g., vitamin A). In addition, it participates in the metabolism of lipids and carbohydrates.

Cirrhosis is often preceded by hepatitis and fatty liver (steatosis), independent of the cause. If the cause is removed at this stage, the changes are still fully reversible.

The pathological hallmark of cirrhosis is the development of scar tissue that replaces normal parenchyma, blocking the portal flow of blood through the organ and disturbing normal function. Recent research shows the pivotal role of the stellate cell, a cell type that normally stores vitamin A, in the development of cirrhosis. Damage to the hepatic parenchyma leads to activation of the stellate cell, which becomes contractile (called myofibroblast) and obstructs blood flow in the circulation. In addition, it secretes TGF-β1, which leads to a fibrotic response and proliferation of connective tissue. Furthermore, it secretes TIMP 1 and 2, naturally occurring inhibitors of matrix metalloproteinases, which prevents them from breaking down fibrotic material in the extracellular matrix.

The fibrous tissue bands (septa) separate hepatocyte nodules, which eventually replace the entire liver architecture, leading to decreased blood flow throughout. The spleen becomes congested, which leads to hypersplenism and increased sequestration of platelets. Portal hypertension is responsible for most severe complications of cirrhosis.

Diagnosis

The gold standard for diagnosis of cirrhosis is a liver biopsy, through a percutaneous, transjugular, laparoscopic, or fine-needle approach. A biopsy is not necessary if the clinical, laboratory, and radiologic data suggests cirrhosis. Furthermore, there is a small but significant risk to liver biopsy, and cirrhosis

itself predisposes for complications due to liver biopsy. Ascites, low platelet count, and spider nevi are useful physical findings

Simplified Document: CIRRHOSIS

Cirrhosis is a result of continuing liver disease marked by replacement of liver tissue by fibrosis (having too much connective tissue), scar tissue and regenerative nodules (lumps that occur as a result of a process in which damaged tissue is reformed), leading to loss of liver function. Scar tissue cannot do what healthy liver tissue does - make protein, help fight infections, clean the blood, help digest food and store energy. Cirrhosis is usually caused by alcohol addiction, hepatitis B and C (other diseases of the liver causing inflammation), and fatty liver disease, but has many other possible causes. Some cases result from an unknown cause, which is called idiopathic.

Ascites (liquid collecting in the belly) is the most common complication of cirrhosis, and is linked with a poor quality of life, higher risk of infection, and a poor long-term outcome. Other possibly dangerous complications are hepatic encephalopathy (which can cause confusion and coma) and bleeding from esophageal varices (abnormally wide veins of the esophagus, which is the tube that carries food, liquids and saliva from the mouth to the stomach). Cirrhosis is generally not reversible, and treatment usually centers on stopping progression and complications. In advanced phases of cirrhosis the only choice is a liver transplant.

The word “cirrhosis” comes from Greek kirrhos meaning yellow, tawny (the light brown to brownish orange color of the diseased liver) plus the English medical word ending -osis. While the disease was known before, it was Rene Laennec who gave it the name “cirrhosis” in his 1819 work in which he also describes the stethoscope.

Pathophysiology

The liver plays a critical role in the chemical change of proteins (for example, albumin, a kind of protein found in blood), clotting factors (proteins that are involved in the blood coagulation process) and complement (a protein that is found in the blood and is important for fighting infection), removing toxins and storage (for example, vitamin A). In addition, it acts in the metabolism of lipids (for example, fats) and sugars.

Cirrhosis often comes after hepatitis and fatty liver (also called steatosis), independent of the cause. If the cause is removed at this stage, the changes are still fully reversible.

The pathological characteristic of cirrhosis is the development of scar tissue that takes the place of the normal working part of the liver, blocking the portal flow of blood through the organ and disturbing normal function. Recent research shows the crucial role of the stellate (or star shaped) cell, a cell type that normally stores vitamin A, in the development of cirrhosis. Damage to the tissue of the liver leads to changes in the stellate cell, which contracts (called myofibroblast) and blocks blood flow in the circulation. In addition, it releases TGF- ß1 which leads to a fibrotic response and growth of connective tissue. Connective tissue is supporting tissue that surrounds other tissues and organs. Furthermore, it releases TIMP 1 and 2, naturally occurring substances that stop matrix metalloproteinases (enzymes or proteins that speed up chemical reactions in the body), which keep them from breaking down fibrotic material in the network of fibers that hold cells together.

The fibrous tissue bands (also called septa) separate liver cell nodes, which eventually replace the entire liver structure, leading to decreased blood flow throughout. The spleen becomes blocked, which leads to an enlarged spleen and increased separation of platelets (which help wounds heal and prevent bleeding by forming blood clots). Portal hypertension (high blood pressure in the vein that carries blood to the liver) is responsible for most serious complications of cirrhosis.

Diagnosis

The gold standard for diagnosis of cirrhosis is a liver biopsy (removal of tissue from the liver for microscopic examination), done through the skin, through the jugular (a vein in your neck that returns blood from your head), through the abdominal wall, or with fine-needle approach. A biopsy is not necessary if the medical examination, blood and urine tests, and radiologic image (such as x-rays or ultrasound) results suggest cirrhosis. Furthermore, there is a small but important risk to liver biopsy, and cirrhosis itself makes one susceptible for complications due to liver biopsy. Ascites, low platelet count, and spider nevi are useful physical findings.
